# Syndecan-3 and TFPI Colocalize on the Surface of Endothelial-, Smooth Muscle-, and Cancer Cells

**DOI:** 10.1371/journal.pone.0117404

**Published:** 2015-01-24

**Authors:** Mari Tinholt, Benedicte Stavik, William Louch, Cathrine Rein Carlson, Marit Sletten, Wolfram Ruf, Grethe Skretting, Per Morten Sandset, Nina Iversen

**Affiliations:** 1 Department of Medical Genetics, Oslo University Hospital and University of Oslo, Oslo, Norway; 2 Department of Haematology and Research Institute of Internal Medicine, Oslo University Hospital, Oslo, Norway; 3 Institute of Clinical Medicine, University of Oslo, Oslo, Norway; 4 Institute for Experimental Medical Research, Oslo University Hospital and University of Oslo, Oslo, Norway; 5 Department of Immunology and Microbial Science, The Scripps Research Institute, La Jolla, California, United States of America; University College London, UNITED KINGDOM

## Abstract

**Background:**

Tissue factor (TF) pathway inhibitor (TFPI) exists in two isoforms; TFPIα and TFPIβ. Both isoforms are cell surface attached mainly through glycosylphosphatidylinositol (GPI) anchors. TFPIα has also been proposed to bind other surface molecules, like glycosaminoglycans (GAGs). Cell surface TFPIβ has been shown to exert higher anticoagulant activity than TFPIα, suggesting alternative functions for TFPIα. Further characterization and search for novel TFPI binding partners is crucial to completely understand the biological functions of cell associated TFPI.

**Methods and Results:**

Potential association of TFPI to heparan sulphate (HS) proteoglycans in the syndecan family were evaluated by knock down studies and flow cytometry analysis. Cell surface colocalization was assessed by confocal microscopy, and native PAGE or immunoprecipitation followed by Western blotting was used to test for protein interaction. Heparanase was used to enzymatically degrade cell surface HS GAGs. Anticoagulant potential was evaluated using a factor Xa (FXa) activity assay. Knock down of syndecan-3 in endothelial,- smooth muscle- and breast cancer cells reduced the TFPI surface levels by 20-50%, and an association of TFPIα to syndecan-3 on the cell surface was demonstrated. Western blotting indicated that TFPIα was found in complex with syndecan-3. The TFPI bound to syndecan-3 did not inhibit the FXa generation. Removal of HS GAGs did not release TFPI antigen from the cells.

**Conclusions:**

We demonstrated an association between TFPIα and syndecan-3 in vascular cells and in cancer cells, which did not appear to depend on HS GAGs. No anticoagulant activity was detected for the TFPI associated with syndecan-3, which may indicate coagulation independent functions for this cell associated TFPI pool. This will, however, require further investigation.

## Introduction

Tissue factor (TF) pathway inhibitor (TFPI) is the endogenous inhibitor of TF-induced blood coagulation, and it exists in two isoforms; TFPIα and TFPIβ. Both isoforms exert anticoagulant activity by binding to the active sites of the TF-factor VIIa (FVIIa) complex and to factor Xa (FXa) [1, 2]. Endothelial cells account for most of the TFPI production, but TFPI expression has also been demonstrated in other cell types, such as monocytes, smooth muscle cells, platelets [3–5], and in several breast cancer cell lines [6, 7].

TFPIα consists of three tandem Kunitz inhibitory domains and a highly positively charged C-terminal end [8], whereas TFPIβ contains the first two Kunitz domains followed by a different C-terminus encoding a glycosylphosphatidylinositol (GPI) attachment signal peptide, directing it to the cell surface [1, 9]. Due to these dissimilarities, TFPIβ is exclusively bound to the cell membrane, while TFPIα can either be secreted to the extracellular environment, or be bound to the cell surface through an as yet unidentified GPI linked molecule, and to some extent to heparan sulfate (HS) proteoglycans (HSPGs) [9–12]. The function of cell surface associated TFPI is not well recognized, however, TFPIβ has been suggested to be responsible for most of the anticoagulant activity on cell surfaces, indicating an alternative role of the cell bound full-length splice variant TFPIα [2]. Further investigation of cell surface associated TFPI and putative binding partner(s) is therefore of key importance to the functional understanding of this molecule.

HSPGs are molecules that protrude from cell membranes or extracellular matrix. There are two major families; the syndecan and glypican families that consist of four and six gene variants, respectively. The expression pattern of the HSPGs is highly regulated in a developmental-, spatial-, and cell type specific manner [13]. Syndecans are transmembrane proteins, whereas glypicans are GPI-anchored proteins lacking direct cytoplasmic connection. Both glypicans and syndecans hold glycosaminoglycans (GAGs), mainly HS chains, that are covalently attached to the protein core [13, 14]. The HS chains are of major functional importance, since they interact with and bind to a broad spectrum of biological effector proteins, like chemokines, growth factors, and extracellular matrix components. Through these interactions, HSPGs participate in many essential cellular actions, such as cell adhesion, proliferation, differentiation and migration. HSPGs can modulate ligand-receptor binding by concentrating cytokines in close vicinity to their high-affinity receptors, or function as co-receptors, thereby promoting efficient signal transduction [14, 15]. HSPGs may also be involved in pathophysiology, including malignancy. Modulations of the sulfation pattern of HS chains have been shown to improve growth factor binding and accelerate proliferation in cancer cells [16].

It has been suggested that HSPGs may act as receptors for the internalization of TFPI-FXa complexes in endothelial cells, contributing to the anticoagulant effect of TFPI [17]. Furthermore, TFPI has been shown to bind to glypican 3 in liver cells [18], and syndecan 4 purified from endothelial cells [19]. The Kunitz domain 3 and the C-terminal end of TFPIα are required for optimal binding to endothelial cell surfaces [20, 21] and hepatoma cells [22], suggesting that only the TFPIα isoform associates with HSPGs.

Since it has been proposed that TFPI might be associated with HSPGs [17–19], we aimed to further characterize the nature of cell bound TFPI. This was performed by evaluating the expression of syndecans 1–4 and the binding potential of endogenously expressed TFPI to syndecans on the cell surface of primary human endothelial- and smooth muscle cells, and basal breast cancer cells.

## Materials and Methods

### Ethics Statement

N/A

### Reagents

Silencer negativ control siRNA #5 (AM4642) was purchased from Ambion (Foster City, C.A, USA). Custom designed siRNAs against syndecans were produced by Eurofins MWG Operon (Ebersberg, Germany) (S1 Table). Rabbit anti-human tissue factor pathway inhibitor IgG 4901/ADG72 and recombinant full length TFPI (ADG49) were obtained from American Diagnostica (Stamford, CT, USA). Goat anti-TFPIα ab9881 and rabbit anti-syndecan-3 ab63932 were from Abcam (Cambridge, MA, USA). Rabbit-IgG-UNLB and goat anti-rabbit IgG(H+L)-RPE were both from Southern Biotechnology Associates (Birmingham, AL, USA). Anti-syndecan-3 (sc-30883), anti-syndecan-3 (sc-9495), the syndecan-3 293T control lysate (sc-176304), normal goat IgG (sc-2028), anti-actin (sc-1616), and A/G agarose beads (sc-2003) were obtained from Santa Cruz Biotechnology, USA. Recombinant syndecan-3 (3539-SD), the secondary antibody anti-goat IgG HRP (HAF109), and Recombinant Human Active Heparanase (7570-GH) were from R&D Systems (Minneapolis, MN, USA). Anti-syndecan-3 1C7 was a kind gift from Dr. Guido David (University of Leuven, Leuven, Belgium). Anti Human D-Heparan Sulfate (F69–3G10 clone, product code 370260–1) was from AMS Biothechnology (Abingdon, UK). The human γ-globulin and Phorbol 12-myristate 13-acetate (PMA) and Heparinase I+III from *Flavobacterium heparinum* (H3917) were from Sigma-Aldrich (St. Louis, MO, USA). Alexa Fluor antibodies (A11071 and A11055) and the transfection reagent Lipofectamine 2000 were from Life Technologies. The commercial enzyme-linked immunosorbent assays (ELISA) Asserachrom Total TFPI and Free TFPI (Diagnostica Stago, Asnière, France) were used to measure TFPI antigen levels, according to the manufacturer’s instructions.

### Cell cultures

The intraductal breast carcinoma cells Sum102 (from the University of Michigan; www.cancer.med.umich.edu/breast_cell/Production/index.html) [6, 7] were grown in HuMEC ready medium (Invitrogen), and the human coronary artery endothelial cells (HCAEC; #CC-2585, Lot no. 6F4194, Lonza) [7] were cultered in EBM-2 (Lonza). All aforementioned cell media with supplements have been described, previously [7]. The human coronary artery smooth muscle cells (HCASMC; #CC-2583, Lot no. 3F0172, Lonza) [23] were grown in SmBM basal medium with SmGM-2 Single Quots growth factors and 10% FCS. Cells were cultured at 37°C in a humidified atmosphere and 5% CO_2_.

### qRT-PCR

Assays for the four syndecans were designed using the Universal Probe Library, Probe Finder software (Roche Applied Sciences). Probe ID and primer sequences are provided in S2 Table. cDNA (19 ng) was amplified in triplicates by quantitative real time PCR (qRT-PCR) with 100 nM probe and 200 nM primers (Eurogentec). An artificial negative control without syndecan expression (Ct = 40), and an endogenous control (18S) Ct value set at the average of all cell types served to calculate the relative mRNA quantity (RQ) of syndecans by the ΔΔCt method. For a more detailed description, please refer to our previous publication [7].

### Small interfering (si)RNA transfection

Based on earlier optimization experiments, cells were transfected with siRNA directed against syndecan 1–4, using Lipofectamine 2000 according to the manufacturer’s recommendations. In short, cells were seeded in 6-well or 12-well plates to reach 30–50% confluence the following day and transfected with 5 or 2.5 μg Lipofectamine and 1 or 0.5 nmol siRNA, in a total volume of 2 or 1 mL fresh medium, respectively. Lipofectamine-containing medium was replaced with fresh medium 5 hours post transfection. Cells were allowed to grow for 2–3 days before analysis.

### Flow cytometry

Cells with transient knock down of syndecans were analysed for cell surface binding of a TFPI specific antibody, essentially as previously described [7]. Differences in TFPI cell surface levels were calculated and presented as percentage reduction compared to control cells (mean values ± SD) based on median fluorescence intensity values. The median fluorescence values for cells labelled with the irrelevant rabbit antibody were considered as unspecific binding, and were corrected for in each individual sample. Cells transfected with negative control siRNA and labelled with irrelevant anti-rabbit antibody served to determine the background level when histogram plots were created (referred to as “irrelevant”). Syndecan-3 cell surface levels were determined similarly using a syndecan-3 specific antibody.

### Heparanase and heparinase treatment

To degrade cell surface HS GAGs, confluent cells in 12-well trays were washed twice with PBS before treated with heparanase (1μg/mL) or heparinase I+III (2U/mL) in serum-free medium (SFM) for 4–16 hours. SFM containing the appropriate vehicle buffer served as a control. After treatment, supernatants were collected, and cells were washed twice before lysis. The effect of heparinase treatment was confirmed by Western blotting using the D-Heparan Sulfate antibody that recognizes HS neo-epitopes generated by digestion of HS by heparinase I+III.

### Confocal microscopy

Cells were seeded on sterile laminin coated glass slides and incubated over night at 37°C. The following day, cells were washed three times in PBS and fixed for 10 min in 4% paraformaldehyde, quenched for 15 min in 100 mM glycine (pH 7.4), and washed three times before blocking for 2 hours in high blocking solution (0.9% NaCl, 0.5% Na_3_Citrate, 3% BSA and 40 ng/μL human γ-globulin) with constant tilting. Subsequently, cells were incubated with 5 μg/mL goat anti-TFPI antibody (ab9881), specific to TFPIα, in a low blocking solution (0.9% NaCl, 0.5% Na_3_Citrate, 1% BSA and 40 ng/uL human γ-globulin) for 4–5 hours. Cells were washed five times in PBS before being incubated for 1.5 hours with 1:1500 Alexa Fluor 488 donkey anti-goat antibody, washed again five times and incubated with 5 μg/mL rabbit anti-syndecan-3 antibody (ab63932) overnight at 4°C under constant tilting. The next day, cells were washed five times, and finally incubated with 1:1500 Alexa Fluor 633 Goat anti-rabbit antibody for 1.5 hours. All antibody incubations were carried out at 4°C. Cells were scanned using an LSM 710 confocal microscope (Zeiss, GmbH, Jena, Germany) with a 40x water immersion objective. The TFPI signal was excited at 488 nm, with emission measured between 500–600 nm. The syndecan-3 signal was excited at 633 nm, with emission measured above 640 nm. Pinhole width was set at 1 Airy unit, and images were collected with an optical slice thickness of 1 μm. Confocal images were de-convolved to compensate for optical distortion by employing Huygen’s Essential software (Scientific Volume Imaging B.V.; Laapersveld, The Netherlands). Images were analyzed and presented using the Image J program (version 1.4.3.67).

### Lysis buffers and immunoprecipitation experiments

Sum102 cells, HCAECs and HCASMCs were harvested in Triton lysis buffer (20 mM HEPES, pH 7.5, 150 mM NaCl, 1 mM EDTA, 0.5% Triton X-100, 0.2% aprotinin, 0.6% 100 mM phenylmethylsulfonylfluoride PMSF, and 1.0% phosphatase inhibitor cocktail) for native (non-reducing) PAGE analysis and for immunoprecipitation experiments (reducing conditions).

2 μg of anti-syndecan-3 or normal goat IgG (negative control) was added to each cell lysate and incubated for immunoprecipitation over night at 4°C. The immunocomplexes were collected by protein A/G agarose beads and washed three times in Triton lysis buffer before boiling in SDS loading buffer followed by SDS-PAGE analysis.

### PAGE analyses and immunoblotting

Protein cell lysates, immunoprecipitates and recombinant TFPI protein (100 ng) were analyzed on precast 4–15% Criterion Tris-HCL gels (#345–0028, BioRad Laboratories) with (reducing) or without 0.1% SDS (non-reducing conditions) in Tris/Glycine/SDS (25 mM Tris, 192 mM Glycine, 0.1% SDS, pH 8.3, #161–0772,) or Tris/Glycine running buffer (25 mM Tris, 192 mM Glycine, pH 8.3, #161–0771, BioRad Laboratories), respectively. A native sample buffer (#161–0738, BioRad Laboratories) was used for non-reducing conditions. Proteins were blotted onto PVDF membranes (RPN 303F, GE Healthcare), which were blocked in 1% casein in TBST for 60 min at room temperature, incubated overnight at 4°C with primary antibodies, washed three times 10 min in TBST and incubated with a horseradish-peroxidase-conjugated secondary antibody. Blots were developed using ECL Prime (RPN 2232, GE HealthCare). The chemiluminescent signals were detected by Las 1000 (Fujifilm, Tokyo, Japan). Immunoblots of immunoprecipitates were stripped for 30 min between antibodies. For native PAGE, two identical immunoblots were run in parallel to ensure specific signals from anti-TFPIα and anti-syndecan-3. Thereafter the immunoblots were stripped for 45 min (Pierce) and reprobed to confirm that the antibodies recognized a protein complex of identical size. ImageJ was used for densitometric quantification of protein bands on immunoblots.

### TF-FVIIa activity

HCAEC and Sum102 cells were transfected with siRNA against syndecan-3 (48 h) in 12-well plates as described above. Cells were washed twice in wash solution (10 mM HEPES, 150 mM NaCl, 4 mM KCl, and 11 mM Glucose, pH 7.5) and incubated for 1 hour at 37°C in reaction solution (wash buffer with 5 mg/mL BSA and 5 mM CaCl_2_, pH 7.5) containing 10 nM FVIIa and 175 nM FX. Following incubation, 50 μL were transferred to 100 μL stop solution (50 mM Tris HCl, 150 mM NaCl, 25 mM EDTA, and 1 mg/mL BSA, pH 7.5) on ice before incubated with 50 μL FXa substrate (CS-11(22)). The absorbance at 405 nm was recorded at 37°C for 45 min at 15 sec intervals using a Spectra Max Plus 384 microplate reader (Molecular Devices, Sunnyvale, CA, USA). The maximum velocities (V_max_) in mU/min were used to calculate the amount of FXa generated, using a standard curve obtained with known concentrations of FXa. HCAEC cells were treated with PMA (10 nM, 6 hours) prior to the assay to induce the TF expression. A ~600-fold upregulation of TF mRNA levels was confirmed using qRT-PCR.

### Statistical methods

Statistical analysis was performed using GraphPad Prism software version 5.0 (PRISM5) (GraphPad Software Inc, LaJolla, CA). Student’s t-tests were performed to test for significant differences when data were normally distributed. Otherwise, the non-parametric Mann Whitney U test was used. *p<0.05, **p<0.01 and ***p<0.001.

## Results

### Expression levels of syndecans in TFPI-expressing cells

**Figure 1:**
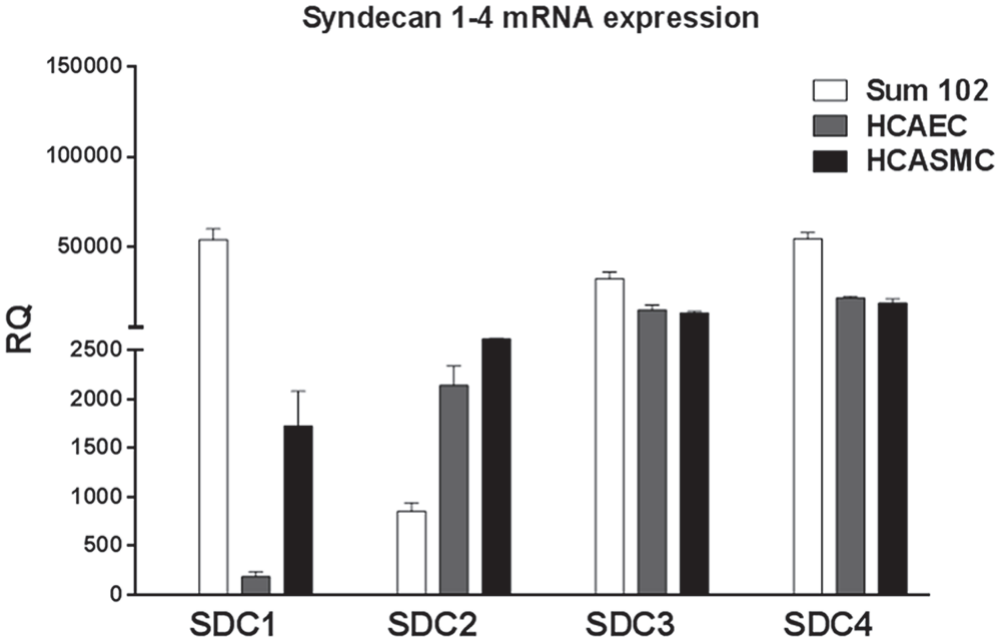
Syndecan 1–4 mRNA expression in Sum102, HCAEC, and HCASMC cells. Sum102, HCAEC, and HCASMC cells were analyzed for mRNA expression of syndecan 1–4 by qRT-PCR. The ΔΔCt method was used to calculate the relative expression (RQ) against the average endogenous control expression among all the cells, and the threshold was set at Ct = 40 (no amplification). Mean values + SD (n = 3 biological parallels) are presented.

HSPGs have previously been reported to bind TFPI. In the search for potential TFPI binding candidates, the mRNA expression levels of syndecans 1–4 were determined in a selection of TFPI-expressing cells. The expression levels of the syndecans varied considerably between the three cell types tested; HCAEC, HCASMC, and Sum102 (Fig. 1). The syndecans were expressed in all cell lines, and the expression levels of syndecan-3 and 4 showed the least variation among the cells.

### Effect of syndecan knock down on the cell surface levels of TFPI

To explore whether the syndecans might be potential TFPI cell surface binding candidates, siRNA technology was applied. After transient knock down of syndecans 1–4, the cells were allowed to bind a TFPI specific antibody followed by staining with a RPE-linked secondary antibody and flow cytometry analysis.

**Figure 2:**
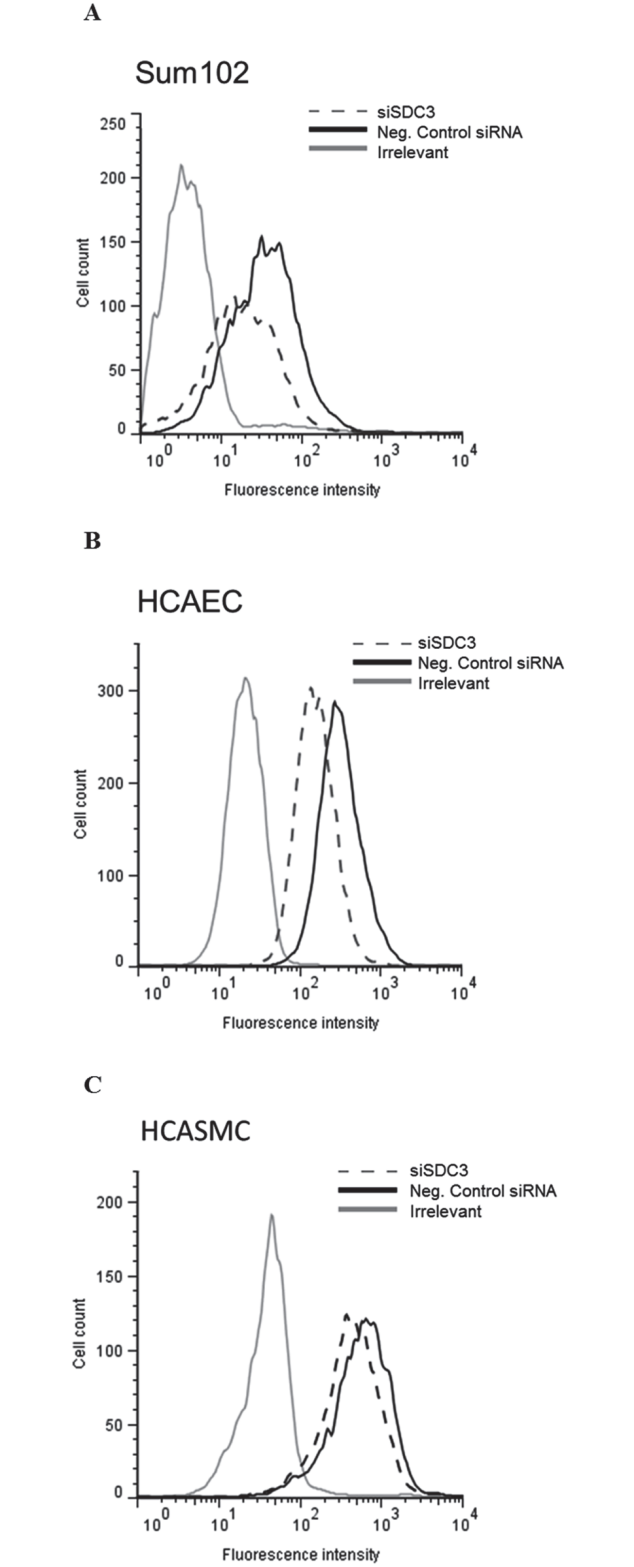
Syndecan-3 knock down reduce the cell surface levels of TFPI. Cells knocked down for syndecan-3 by siRNA technology were analyzed for levels of cell surface TFPI by flow cytometry. The histogram presents median fluorescence intensity obtained after TFPI specific antibody labelling in A) Sum102 cells, B) HCAEC cells and C) HCASMC cells. Syndecan-3 knocked down cells (siSDC3); dashed line, negative control siRNA; black solid line and irrelevant control; grey solid line. One representative experiment of three individual experiments (n≥6 biological parallels) is shown for each cell type.

**Figure 3:**
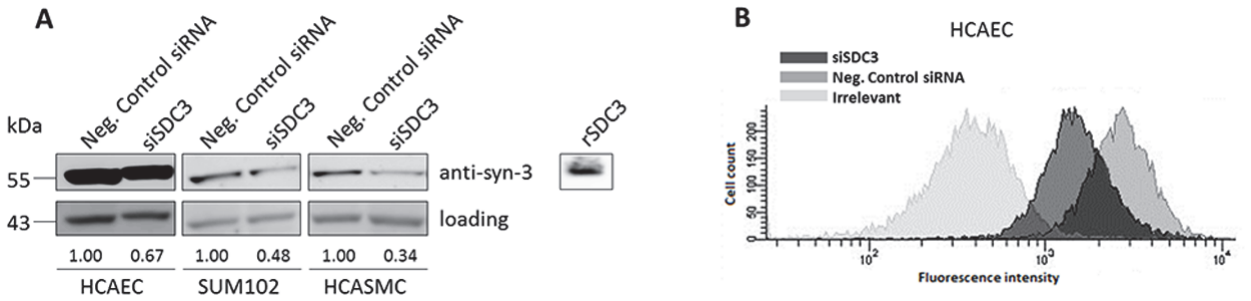
Syndecan-3 antigen levels in syndecan-3 knocked down cells. A) Syndecan-3 antigen levels (the 55 kDa monomeric form) in cell lysates from HCAECs Sum102 cells, and HCASMCs by Western blotting using anti-syndecan-3 and loading control (actin). One representative membrane of two is shown. Syndecan-3 protein band intensities (after normalization against loading control) relative to control cells are indicated for each cell type. Recombinant syndecan-3 (rSDC3) served as a positive control (~110 kDa). B) Cell surface associated syndecan-3 levels analyzed by flow cytometry in HCAEC cells. The histogram presents median fluorescence intensity obtained after syndecan-3 specific antibody labelling. Syndecan-3 knocked down cells (siSDC3); dark grey shaded, negative control siRNA; medium grey shaded and irrelevant control; light grey shaded. One representative experiment of two individual experiments (n = 4 biological parallels) is shown.

No significant decrease in cell surface levels of TFPI was observed for any of the cell types after knock down of syndecan 1, 2 or 4 (data not shown). In contrast, syndecan-3 knock down in the HCAECs, HCASMCs, and Sum102 cells decreased the cell surface levels of TFPI by 52 ± 9%, 23 ± 6%, and 39 ± 12% (mean ± SD), respectively (Fig. 2). Knock down efficiencies of the syndecans in siRNA transfected cells were confirmed by qRT-PCR and calculated as percent knock down compared to control cells transfected with negative control siRNA. The knock down efficiencies varied between 69–97% for syndecan 1, 45–85% for syndecan 2, 83–95% for syndecan-3, and 44–72% for syndecan 4 (S1 Fig.). Furthermore, the syndecan-3 knock down was confirmed by Western blotting in all cell types (Fig. 3A), and a 32 ± 13% (mean ± SD) reduction in syndecan-3 antigen levels at the cell surface after syndecan-3 knock down was demonstrated by flow cytometry in HCAEC (Fig. 3B).

Neither heparanase (S2 Fig.) nor heparinase I+III (data not shown) treatment of Sum102, HCAEC and HCASMC cells changed the TFPI antigen levels in cell supernatants compared to control cells.

### Effect of TFPI knock down on the syndecan-3 mRNA expression

**Figure 4:**
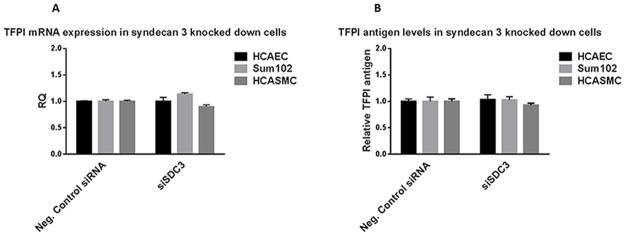
TFPI mRNA and antigen levels in syndecan-3 knocked down cells. A) HCAEC, Sum102 and HCASMC cells with syndecan-3 knocked down were analysed for TFPI mRNA expression by qRT-PCR. The ΔΔCt method was used to calculate the relative TFPI expression (RQ) compared to control cells (Neg. Control siRNA). Mean values + SD (n≥6 biological parallels) of three individual experiments are presented. B) TFPI antigen levels were measured by ELISA in cell lysates from HCAEC, Sum102 and HCASMC after syndecan-3 knock down. TFPI antigen levels relative to control cells are shown. Mean values + SD (n≥6 biological parallels) of two individual experiments are presented.

**Figure 5:**
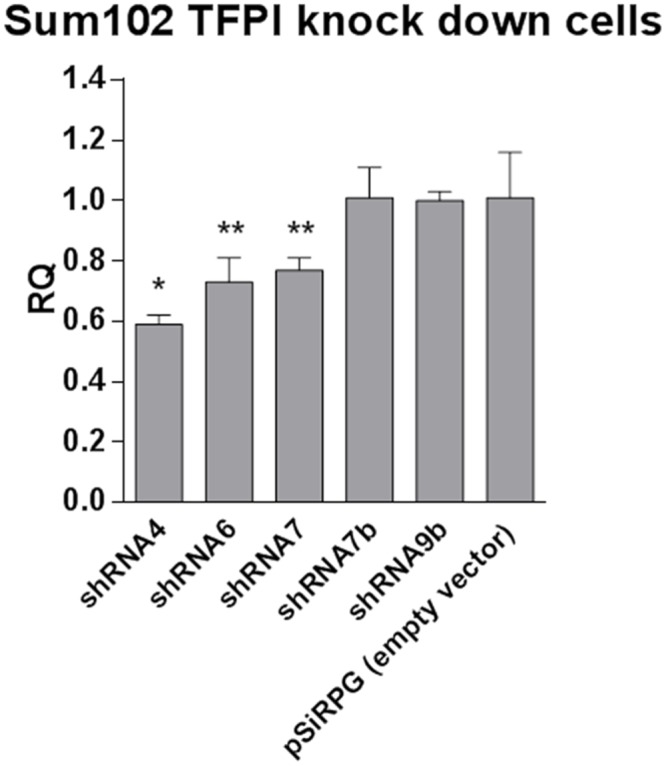
Syndecan-3 mRNA expression in Sum102 TFPI knock down cells. Syndecan-3 mRNA expression was measured by qRT-PCR in three independent stable clones with both isoforms of TFPI (α+β) knocked down (shRNA 4, 6 and 7) and two independent stable clones with only the TFPIβ isoform knocked down (shRNA7β and 9β). Results were normalized against endogenous control and relative expressions (RQ) were calculated in reference to the empty vector control cells (pSiRPG). Mean values + SD (n = 3 biological parallels) are presented.

Knock down of syndecan-3 did not affect the TFPI mRNA expression (Fig. 4A) or the TFPI protein levels (Fig. 4B). Nevertheless, experiments were conducted to assess whether a unidirectional regulatory mechanism existed. The syndecan-3 mRNA expression was reduced by a mean of 30% in three separate pools of Sum102 cells, in which both TFPI isoforms were stably knocked down (the TFPI knock down efficiencies were 40–60%, as described in Stavik *et al*. 2011 [6]). No differences in syndecan-3 mRNA expression levels were detected when only the TFPIβ isoform was knocked down (Fig. 5) (the TFPIβ knock down efficiencies were 53% and 64% for shRNA7b and shRNA 9b, respectively). In HCAEC and HCASMC cells, a 48% and 43% reduction of syndecan-3 mRNA expression was detected in cells with 88–94% transient TFPI (α+β) knock down (48 hours). Furthermore, knock down of the TFPIα isoform alone (TFPIβ expression remained unaffected) in Sum102 cells (72 hours) and HCAECs (48 hours) resulted in a respective 34% and 53% reduction of syndecan-3 mRNA expression. The TFPI knock down efficiencies of all cell types used are shown in S3 Fig.

### TFPI and syndecan-3 colocalization

**Figure 6:**
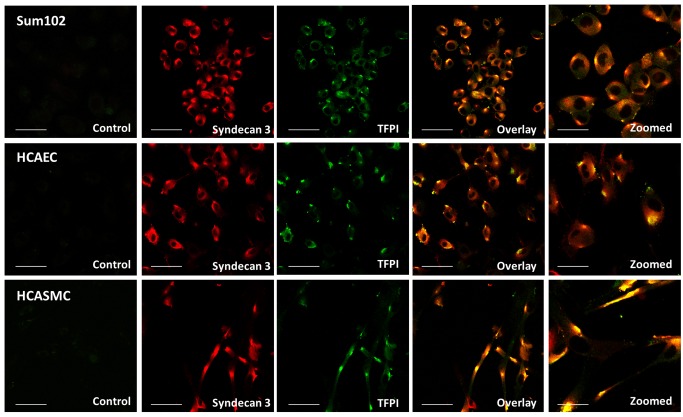
TFPI and syndecan-3 colocalize at the cell surface. Fixed cells were double stained with TFPI (green) and syndecan-3 (red) primary antibodies and Alexa Fluor secondary antibodies with 488 and 633 nm excitation wavelengths, respectively, before images were captured using confocal microscopy. Yellow colour in the overlay images demonstrate spatial overlap between TFPI and syndecan-3. Sum102 cells (top), HCAEC cells (middle) and HCASMC cells (bottom). Scale bar 50 μM (30 μM for zoomed images). One representative experiment of three individual experiments is shown for each cell type.

To further investigate if the reduced binding of cell surface TFPI was due to a direct effect of syndecan-3 knock down, we examined whether TFPIα and syndecan-3 colocalized on the cell surface. Colocalization was confirmed using double staining and confocal microscopy in Sum102, HCAEC, and HCASMC cells. The colocalization was not uniformly distributed, rather concentrated to confined sites of the cell membrane (Fig. 6, S4 Fig., and S5 Fig.).

### Identification of a TFPI-syndecan-3 protein complex

**Figure 7:**
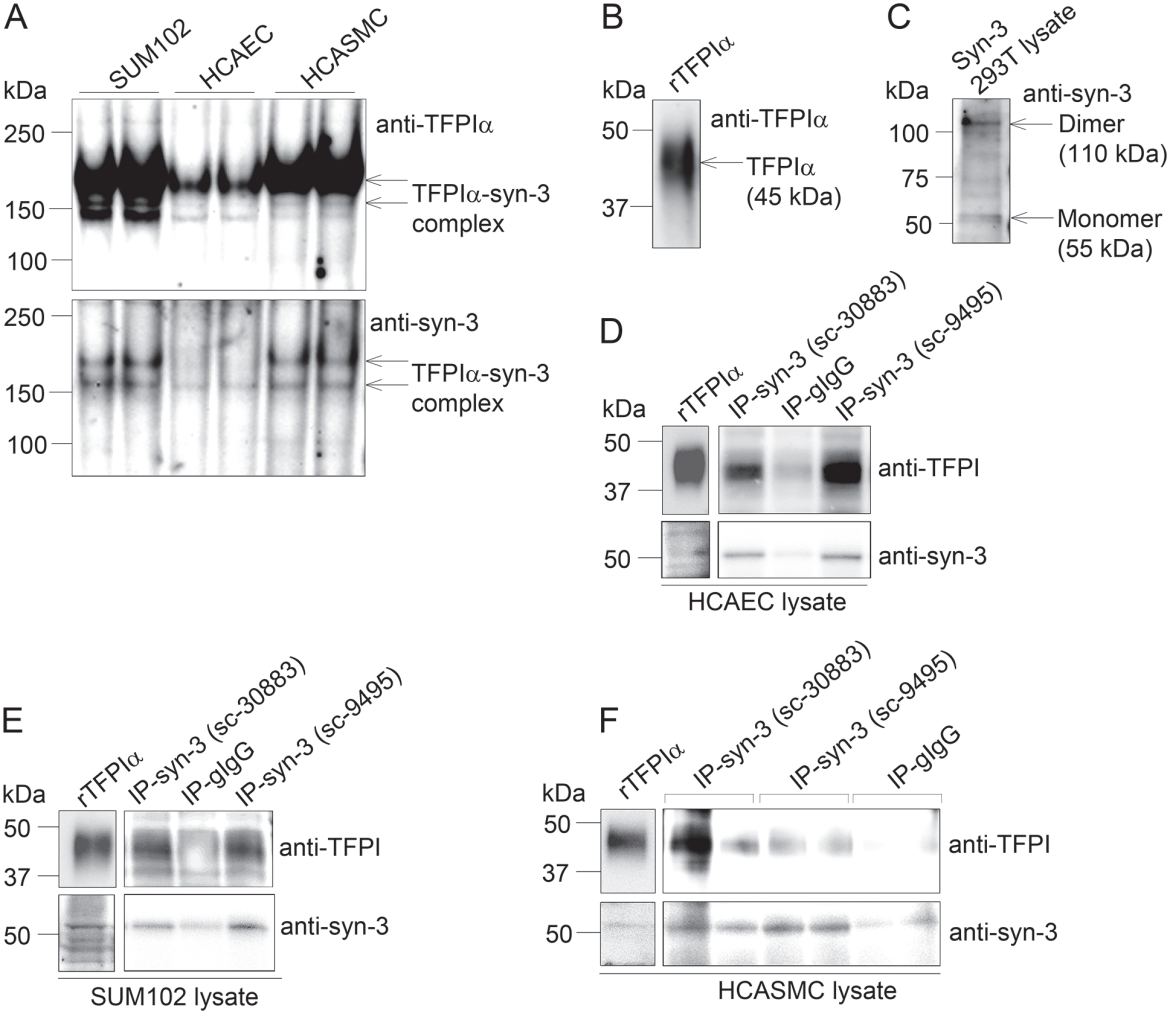
Identification of a TFPI-syndecan-3 protein complex by native PAGE and immunoprecipitation. Protein lysates were isolated from cells, subjected to native PAGE analysis or immunoprecipitation experiments and analysed by Western blotting. A) Native PAGE of lysates from Sum102 cells (20 μg), HCAECs (12 μg) and HCASMCs (18 μg) immunoblotted with anti-TFPIα (top) and anti-syndecan-3 (bottom) (one representative membrane out of three is shown), B) SDS-PAGE of full length recombinant TFPIα protein immunoblotted with anti-TFPIα, and C) SDS-PAGE of syndecan-3 293T control lysate immunoblotted with anti- syndecan-3. D) HCAEC (40 μg), E) SUM102 (160 μg) and F) HCASMC (40 μg) lysates were subjected to immunoprecipitation using two different syndecan-3 antibodies (n = 2 for sc-30883, n = 1 for sc-9495) or control goat IgG (n = 2). Lysates and immunoprecipitates were analysed for the presence of endogenous syndecan-3 and TFPI by immunoblotting. Recombinant TFPIα protein (upper left panel) and cell lysate (lower left panel) was used as positive control for the immunoblotting in D-F.

Although the confocal imaging suggested that TFPIα and syndecan-3 resided at the same physical location (Fig. 6, S4 Fig., and S5 Fig.), native PAGE followed by subsequent Western blotting was performed to test for an interaction between the two molecules (Fig. 7A). In protein lysates from Sum102, HCAEC, and HCASMC cells, two protein complexes migrating at apparent masses of ~150 kDa and ~180 kDa were recognized both by anti-TFPIα and anti-syndecan-3 (Fig. 7A). The two complexes most likely result from different post-translational modifications such as glycosylation. Weaker bands were observed for HCAEC cells, reflecting the lower protein concentrations applied. The relatively large sizes of the observed complexes indicated that TFPIα (Fig. 7B, 45 kDa) was present with the dimerized form of syndecan-3, which has an approximate size of 110kDa (Fig. 7C). The size estimations should, however, be interpreted with some caution since non-reducing (native) conditions were used and also because precise separation of high molecular weight complexes by electrophoresis in general is challenging.

The TFPI-syndecan-3 interaction was also analysed by immunoprecipitation. Immunoprecipitation of syndecan-3 from HCAEC lysate using two different syndecan-3 antibodies revealed co-precipitation of TFPI (Fig. 7D), suggesting that TFPI associated with syndecan-3. Consistent with the native PAGE analysis (Fig. 7A), the TFPI-syndecan-3 complex was present also in SUM102 (Fig. 7E) and HCASMC lysate (Fig. 7F), although the TFPI precipitation appeared somewhat weaker.

### TFPI distribution following syndecan-3 knock down

**Figure 8:**
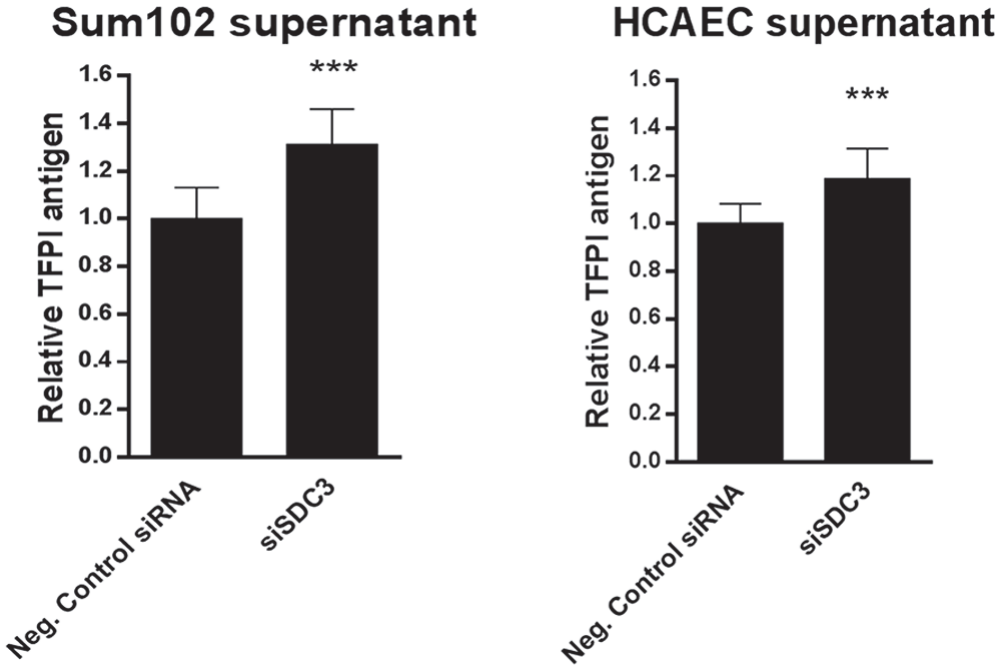
TFPI antigen levels in supernatant from Sum102 and HCAEC cells after syndecan-3 knock down. TFPI antigen levels (total) were measured by ELISA in supernatants from Sum102 (left) and HCAEC (right) after syndecan-3 knock down. Relative TFPI antigen levels compared to control cells are shown. Mean values + SD (n≥9 biological parallels) of two (HCAEC) and three (Sum102) individual experiments are presented.

In order to establish the fate of TFPI in syndecan-3 deficient cells, TFPI antigen was measured by ELISA in the supernatant of Sum102 and HCAEC cells after knock down of syndecan-3. This resulted in a 20–30% increase of TFPI antigen levels in the supernatant compared to control cells (Fig. 8).

### TF-FVIIa activity at the surface of syndecan-3 knock down cells

**Figure 9:**
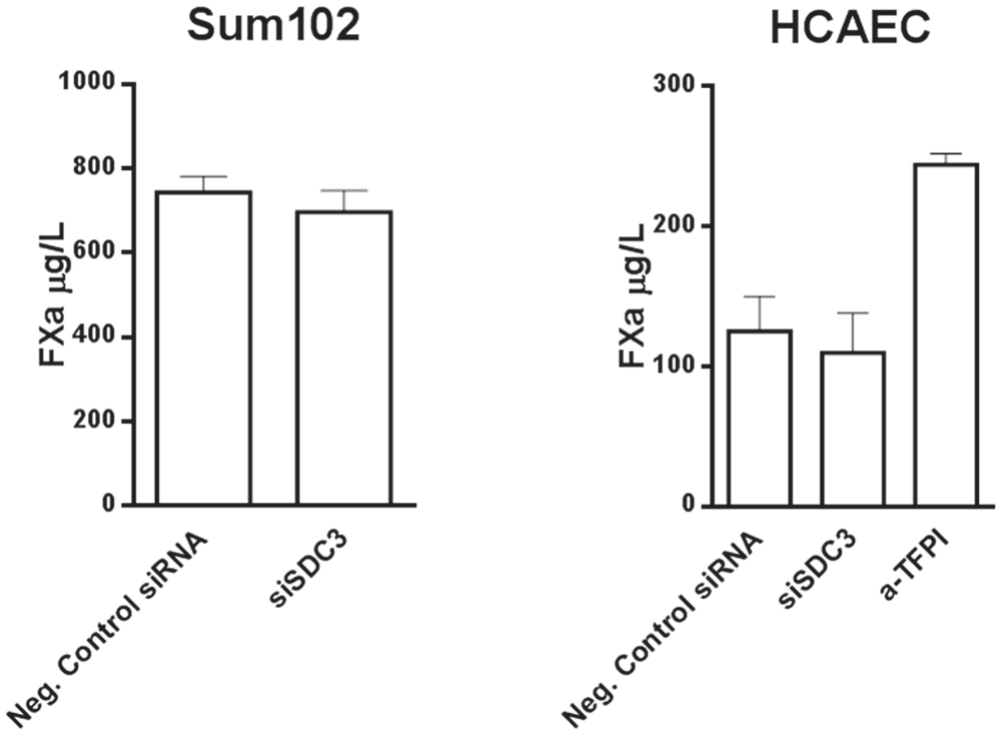
TF-FVIIa cell surface activity in Sum102 and HCAEC cells after syndecan-3 knock down. Sum102 and HCAEC cells knocked down of syndecan-3 (siSDC3) were analyzed for TF-FVIIa cell surface activity, indirectly, as FXa generation. HCAEC cells were stimulated with 10 nM PMA for 6 hours prior to analysis. Anti-TFPI was added to one experiment with HCAEC cells. Mean values + SD (n≥8 biological parallels) of three individual experiments are presented.

To explore whether TFPI bound to syndecan-3 could arrest TF coagulant activity, we measured the TF-FVIIa activity at the surface of Sum102 and HCAEC cells before and after transient knock down of syndecan-3. TF-FVIIa activity was determined indirectly by quantification of FXa generated in a colorimetric assay where FVIIa and FX were exogenously added to adherent cells. No changes in the FXa generation after syndecan-3 knock down were observed in any of the two cell types (Fig. 9). As expected, a ~2-fold increase in TF-FVIIa activity was observed upon addition of anti-TFPI to the assay (positive control).

## Discussion

In the present study, we have demonstrated an association of endogenously expressed TFPI to the transmembrane syndecan-3 HSPG molecule. Our approach has been to knock down the expression of HSPGs belonging to the syndecan family, and to analyse the TFPI antigen surface levels by flow cytometry. We selected endothelial-, smooth muscle-, and breast cancer cells with high endogenous TFPI expression for investigation. The syndecan expression levels varied between these cell types in accordance to the fact that HSPG expression differs in a cell type- and developmental specific manner [13, 24]. Reduced cell surface levels of TFPI were detected by flow cytometry after syndecan-3 knock down in all three cell types, indicating involvement of syndecan-3 in cell surface association of TFPI. Moreover, the decreased syndecan-3 mRNA expression in HCAECs and Sum102 cells with TFPIα knocked down, and in Sum 102 cells with both TFPI isoforms (α+β) knocked down, but not TFPIβ alone, further supports a relation between syndecan-3 and TFPI, specifically the TFPIα isoform. Whether this is a direct or indirect regulation remains to be elucidated.

Previously, syndecan 4 purified from EA.hy926 cells has been shown to bind TFPI in a solid phase assay [19]. To the best of our knowledge, we hereby provide the first report on TFPI association to a syndecan molecule on the cell surface. With a TFPIα specific antibody, colocalization of TFPIα and syndecan-3 was confirmed by confocal microscopy in Sum 102, HCAEC, and HCASMC, and native PAGE indicated that TFPIα was found in complex with syndecan-3. Additionally, an association between TFPI and syndecan-3 was further demonstrated by immunoprecipitation. These experiments demonstrate a GPI-independent anchoring of TFPI to cell surfaces, which may partly explain why phosphatidylinositol-specific phospholipase C (PI-PLC) has been shown to be incapable of removing TFPI completely from the cell surface [7, 9]. Hence, TFPIα exerts flexibility by being associated to the cell surface in both a GPI-dependent and independent manner.

It remains to be elucidated whether TFPIα attaches to its binding partners posttranslationally or after cell secretion. In this study, we learned that a portion of TFPI was released to the external environment in the absence of syndecan-3. This observation may suggest that TFPI binds syndecan-3 extracellularly. The molecular signal for this is at present unknown. As could be expected from the fact that syndecan-3 is not the major binding partner of TFPI, the release of TFPI to the supernatant was moderate, but yet significant.

Attempting to identify whether TFPI binds to the syndecan-3 core and/or to the HS GAGs, we used heparanase or heparinase I+III treatment to remove HS GAG chains from the cells. It has previously been hypothesized that the positively charged C-terminal end of TFPIα is bound through electrostatic forces to negatively charged GAGs on endothelial surfaces. The mechanism by which heparin releases TFPI from endothelial cells was simply thought to be displacement [25]. However, more recent studies have demonstrated that the concentration of TFPI on the cell surface was unaffected by heparin treatment [26–29]. Hence, the GPI-independent cell surface association of TFPIα seems to be more complex than first anticipated. In the present study, the removal of HS chains did not release TFPI antigen from the cells, suggesting that TFPIα surface association does not involve HS chains. This finding is consistent with two other studies reporting that HS removal by heparinase III in HUVECs and EA.hy926 cells neither influenced TFPI antigen level or activity in the supernatant [30], nor binding of exogenously added rTFPI [31]. Nadir et al. found that treatment with their in house produced heparanase led to dissociation of TFPI from the surface of endothelial cells. However, this effect was independent of heparanase enzymatic activity and interaction with HS [32]. Thus, it appears that the TFPI-syndecan-3 interaction observed in our study is not likely to involve HS GAGs.

We have previously reported that a cell surface TFPI pool, not bound through GPI, could inhibit TF-induced coagulation activity [7]. Encouraged by this observation, we tested the anticoagulant potential of Sum102 and HCAEC cells after syndecan-3 knock down, and found that the knock down did not change the cells’ ability to generate FXa. These results are in line with Piro and Broze, who suggested alternative roles for cell surface bound TFPIα in endothelial like cells. They, as well as Maroney and coworkers found that TFPIα was less efficient than TFPIβ in inhibiting TF induced coagulation [2, 33]. This opens for a novel role for this type of cell surface bound TFPIα, such as interference with the ability of syndecan-3 to bind cognate ligands. Syndecan-3 is thought to act as a co-receptor for growth factors and extracellular matrix components like basic fibroblast growth factor, heparin binding growth-associated molecule, epidermal growth factor receptor and notch signalling ligands [34–37].

A pilot experiment conducted in the human embryonic kidney cells HEK293T indicated that in addition to syndecan-3, the GPI-anchored glypican 3 molecule could be involved in TFPI binding since a reduction of cell surface associated TFPI antigen was observed in glypican 3 knocked down cells (data not shown). This observation is in agreement with a study by Mast *et al* that previously showed binding of TFPI to glypican 3 in the HepG2 tumor liver cell line [18]. HepG2 and HEK293T cells relate in terms of function, since liver and kidney are both filtration organs. The TFPI-glypican 3 interaction might represent a mechanism for clearing the bimolecular TFPI-FXa complex from the circulation [17]. Glypican 3 expression was not detectable in HCASMC and HCAEC, and only slightly expressed in Sum102 and (0.2% of the glypican 3 expression in HEK293T). Glypican 3 knock down did not alter the cell surface TFPI levels in Sum102, thus demonstrating glypican 3 as an unlikely binding molecule for TFPI in these breast cancer cells.

In conclusion, we provide the first evidence of an association between TFPIα and the GPI-independent syndecan-3 molecule at cell surfaces, including cancer cells. The association did not appear to involve HS chains. Moreover, we could not detect any anticoagulant activity of TFPI bound to syndecan-3, possibly suggesting non-hemostatic roles for this pool of cell surface associated TFPI. However, further ascertainment is warranted, and the specific functional implications of TFPI binding to syndecan-3 needs to be addressed in more detail in future studies.

## Supporting Information

S1 Fig Syndecan 1–4 knock down in Sum102 cells, HCAECs, and HCASMCs. Sum102 cells, HCAECs, and HCASMCs knocked down for syndecan 1–4 by siRNA technology were analyzed for mRNA expression of syndecan 1–4 by qRT-PCR. Results were normalized against endogenous control and relative expressions (RQ) were calculated in reference to the negative control siRNA (Neg. Control siRNA). Mean values + SD (n≥3 biological parallels) of three individual experiments are presented.
(PDF)

S2 Fig TFPI antigen levels in supernatant from Sum102 and HCAEC cells after heparanase treatment. Total TFPI (black bars) and free TFPI (open bars) antigen levels were measured by ELISA in supernatants from A) Sum102, B) HCAEC, and C) HCASMC after treatment with (+) or without (-) heparanase. Relative TFPI antigen levels compared to control cells are shown. Mean values + SD (n≥3 (HCAEC), n≥6 (Sum102), and n = 3 (HCASMC) biological parallels) of two (HCAEC and HCASMC) and three (Sum102) individual experiments are presented.
(PDF)

S3 Fig TFPI knock down in Sum102, HCAEC, and HCASMC cells. A) Total TFPI, TFPIα or TFPIβ mRNA expression was measured by qRT-PCR in A) three independent stable clones with both isoforms of TFPI (α+β) knocked down (shRNA 4, 6 and 7) and two independent stable clones with only the TFPIβ isoform knocked down (shRNA7β and 9β), B) HCAECs (left) and HCASMCs (right) with both isoforms of TFPI (α+β) transiently knocked down by two separate TFPI specific siRNAs (48 hours after transfection), and C) HCAECs (left) and Sum102 cells (right) with only the TFPIα isoform transiently knocked down by two TFPI specific siRNAs in combination (48 and 72 hours after transfection, respectively). Results were normalized against endogenous control and relative expressions (RQ) were calculated in reference to control cells (empty vector (pSiRPG) or Neg. Control siRNA, respectively). Mean values + SD (n = 3 biological parallels) are presented.
(PDF)

S4 Fig TFPI and syndecan-3 colocalize at the cell surface (supplemental to Fig. 6). Fixed cells were double stained with TFPI (green) and syndecan-3 (red) primary antibodies and Alexa Fluor secondary antibodies with 488 and 633 nm excitation wavelengths, respectively, before images were captured using confocal microscopy. Yellow colour in the overlay images demonstrates spatial overlap between TFPI and syndecan-3. Sum102 cells (top), HCAEC cells (middle) and HCASMC cells (bottom). Scale bar 50 μM. Experiment two of three individual experiments is shown for each cell type.
(TIF)

S5 Fig TFPI and syndecan-3 colocalize at the cell surface (supplemental to Fig. 6). Fixed cells were double stained with TFPI (green) and syndecan-3 (red) primary antibodies and Alexa Fluor secondary antibodies with 488 and 633 nm excitation wavelengths, respectively, before images were captured using confocal microscopy. Yellow colour in the overlay images demonstrates spatial overlap between TFPI and syndecan-3. Sum102 cells (top), HCAEC cells (middle) and HCASMC cells (bottom). Scale bar 50 μM. Experiment three of three individual experiments is shown for each cell type.
(TIF)

S1 Table Sequences of siRNA directed against syndecans. (PDF)

S2 Table Probe ID and primer sequences of syndecans (SDC). (PDF)
