# Supplementary figures and images for: Syndecan-3 and TFPI Colocalize on the Surface of Endothelial-, Smooth Muscle-, and Cancer Cells
Source: PLoS One. 2015 Jan 24;10(1):e0117404. doi: 10.1371/journal.pone.0117404 (PMC4305309; doi:10.1371/journal.pone.0117404)

### Syndecan 1-4 knock down

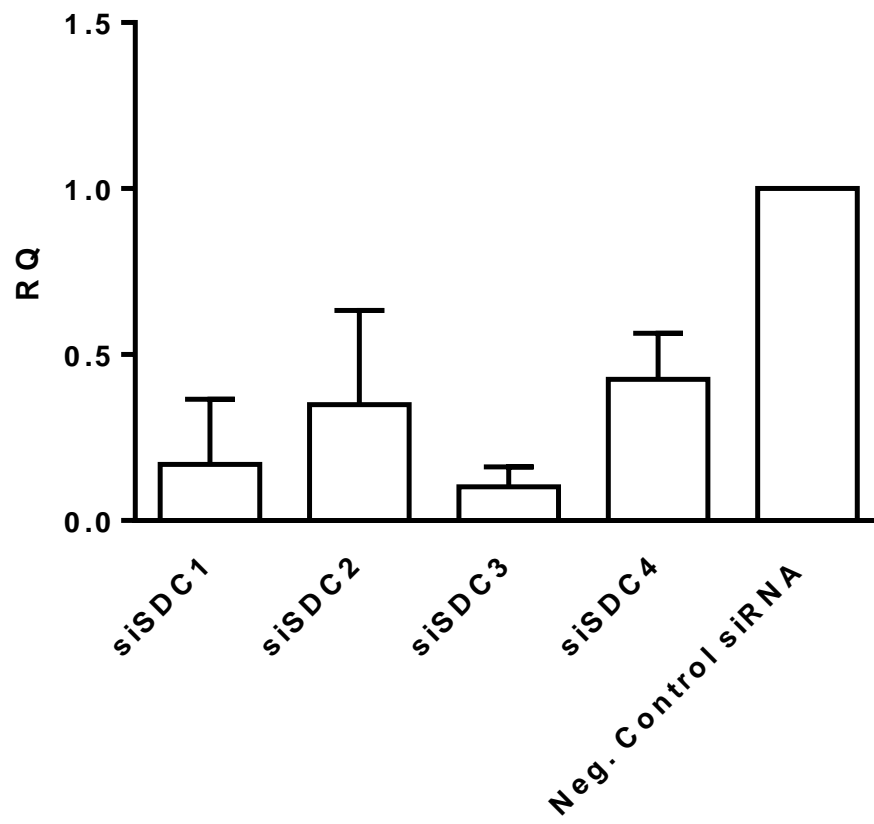

Supplement: S1 Fig — Sum102 cells, HCAECs, and HCASMCs knocked down for syndecan 1–4 by siRNA technology were analyzed for mRNA expression of syndecan 1–4 by qRT-PCR. Results were normalized against endogenous control and relative expressions (RQ) were calculated in reference to the negative control siRNA (Neg. Control siRNA). Mean values + SD (n≥3 biological parallels) of three individual experiments are presented. (PDF) [file pone.0117404.s001.pdf]

**A**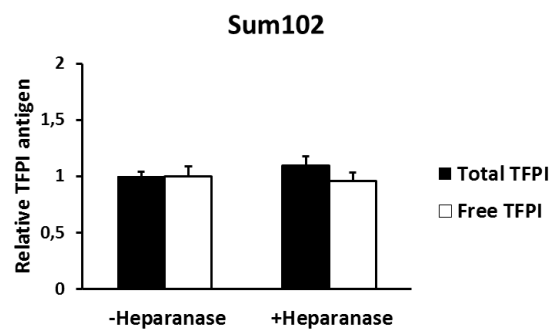**B**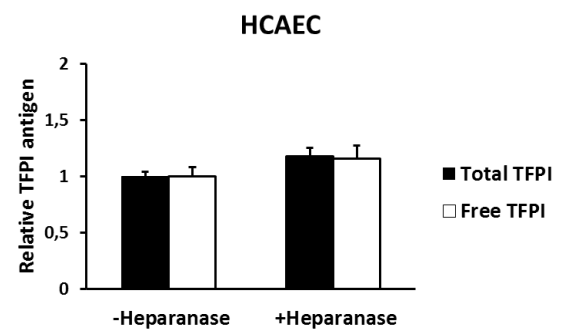**C**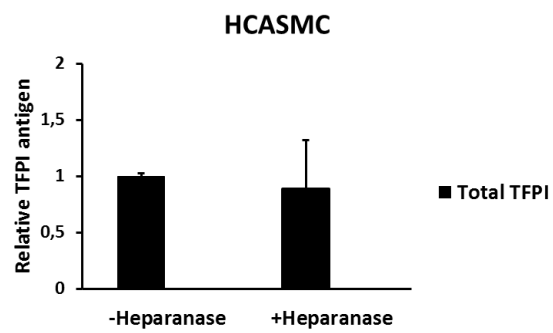

Supplement: S2 Fig — Total TFPI (black bars) and free TFPI (open bars) antigen levels were measured by ELISA in supernatants from A) Sum102, B) HCAEC, and C) HCASMC after treatment with (+) or without (-) heparanase. Relative TFPI antigen levels compared to control cells are shown. Mean values + SD (n≥3 (HCAEC), n≥6 (Sum102), and n = 3 (HCASMC) biological parallels) of two (HCAEC and HCASMC) and three (Sum102) individual experiments are presented. (PDF) [file pone.0117404.s002.pdf]

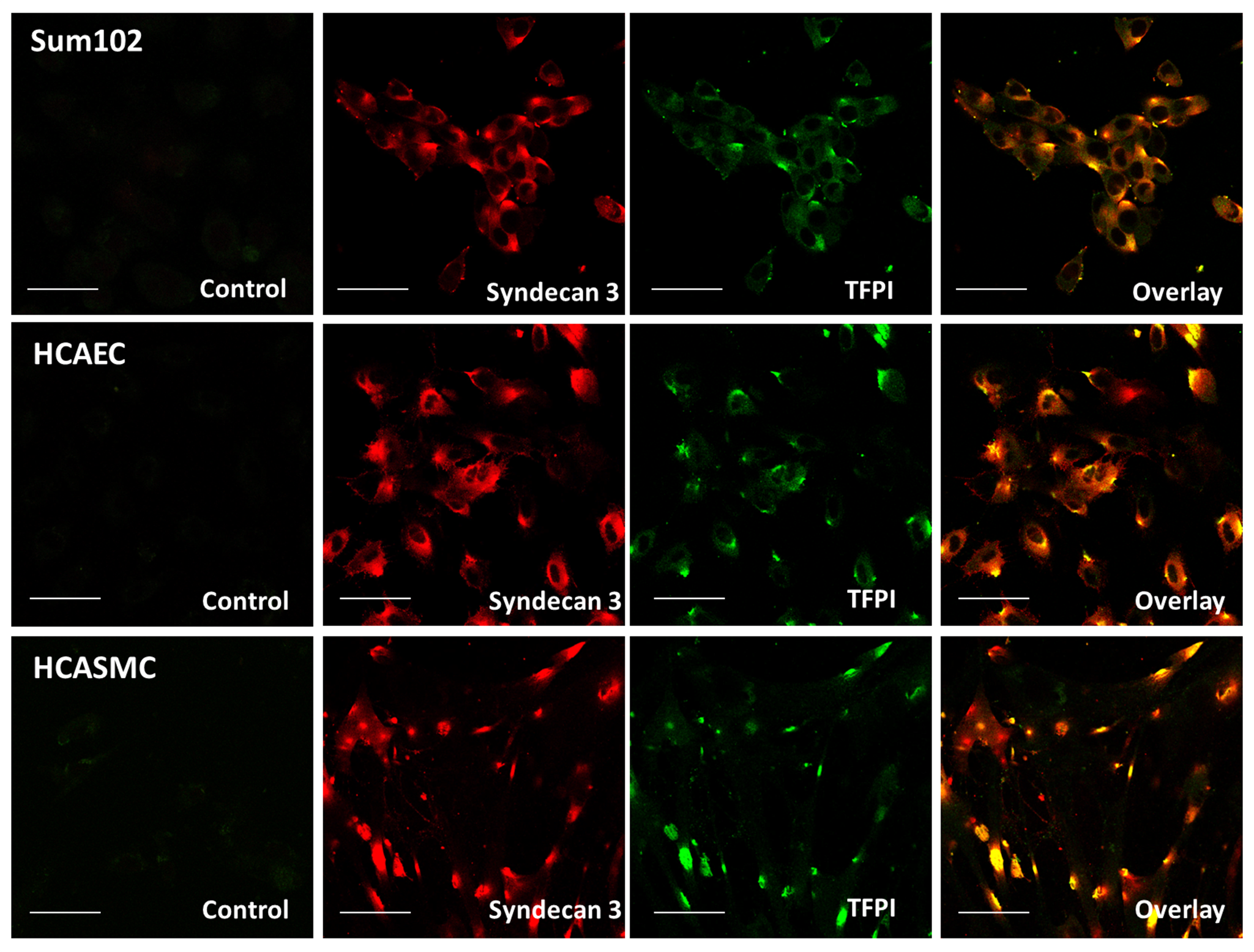

Supplement: S4 Fig — Fixed cells were double stained with TFPI (green) and syndecan-3 (red) primary antibodies and Alexa Fluor secondary antibodies with 488 and 633 nm excitation wavelengths, respectively, before images were captured using confocal microscopy. Yellow colour in the overlay images demonstrates spatial overlap between TFPI and syndecan-3. Sum102 cells (top), HCAEC cells (middle) and HCASMC cells (bottom). Scale bar 50 μM. Experiment two of three individual experiments is shown for each cell type. (TIF) [file pone.0117404.s004.tif]

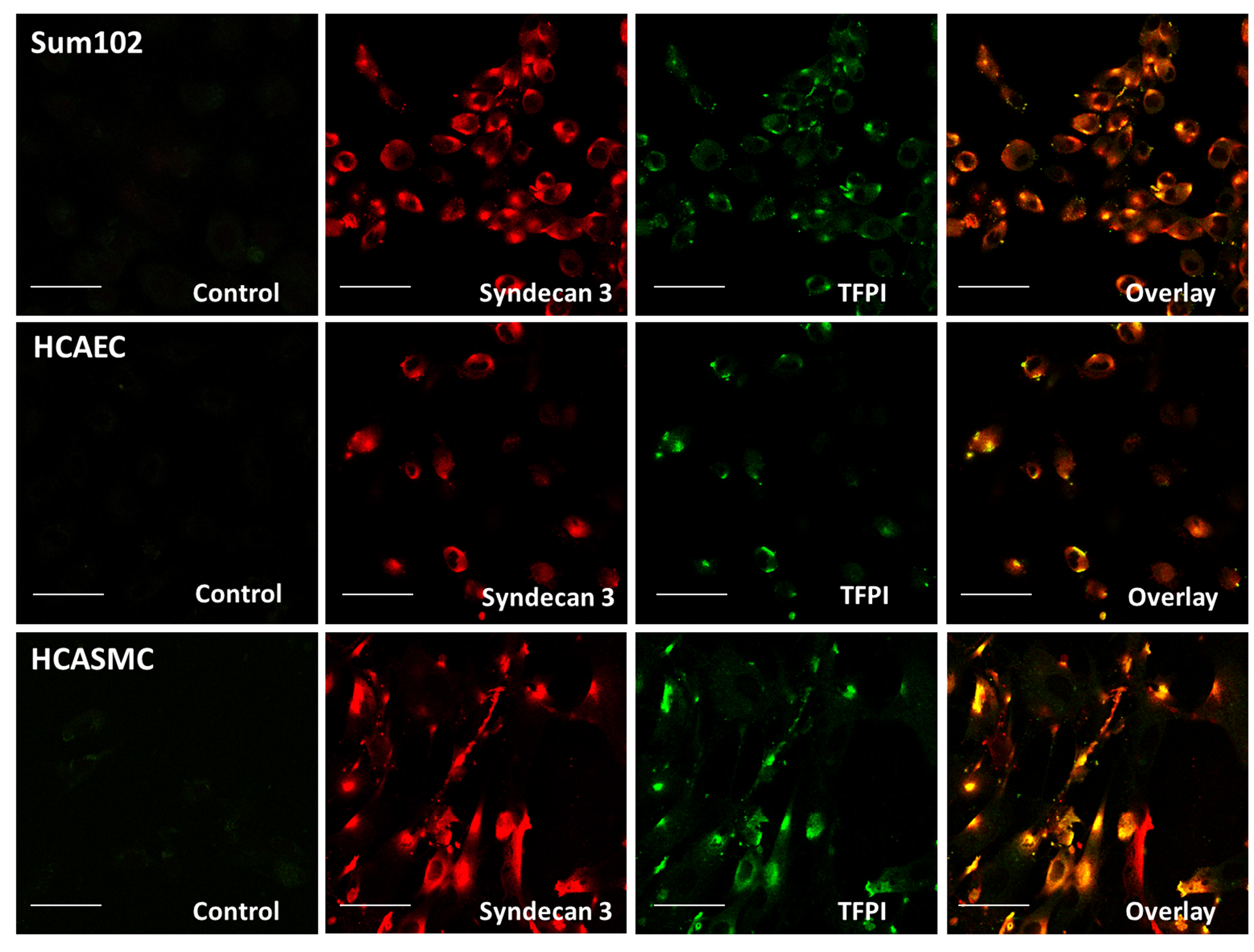

Supplement: S5 Fig — Fixed cells were double stained with TFPI (green) and syndecan-3 (red) primary antibodies and Alexa Fluor secondary antibodies with 488 and 633 nm excitation wavelengths, respectively, before images were captured using confocal microscopy. Yellow colour in the overlay images demonstrates spatial overlap between TFPI and syndecan-3. Sum102 cells (top), HCAEC cells (middle) and HCASMC cells (bottom). Scale bar 50 μM. Experiment three of three individual experiments is shown for each cell type. (TIF) [file pone.0117404.s005.tif]
