# Supplementary material for: Syndecan-3 and TFPI Colocalize on the Surface of Endothelial-, Smooth Muscle-, and Cancer Cells
Source: PLoS One. 2015 Jan 24;10(1):e0117404. doi: 10.1371/journal.pone.0117404 (PMC4305309; doi:10.1371/journal.pone.0117404)
Supplement: S3 Fig — A) Total TFPI, TFPIα or TFPIβ mRNA expression was measured by qRT-PCR in A) three independent stable clones with both isoforms of TFPI (α+β) knocked down (shRNA 4, 6 and 7) and two independent stable clones with only the TFPIβ isoform knocked down (shRNA7β and 9β), B) HCAECs (left) and HCASMCs (right) with both isoforms of TFPI (α+β) transiently knocked down by two separate TFPI specific siRNAs (48 hours after transfection), and C) HCAECs (left) and Sum102 cells (right) with only the TFPIα isoform transiently knocked down by two TFPI specific siRNAs in combination (48 and 72 hours after transfection, respectively). Results were normalized against endogenous control and relative expressions (RQ) were calculated in reference to control cells (empty vector (pSiRPG) or Neg. Control siRNA, respectively). Mean values + SD (n = 3 biological parallels) are presented. (PDF) [file pone.0117404.s003.pdf]

A

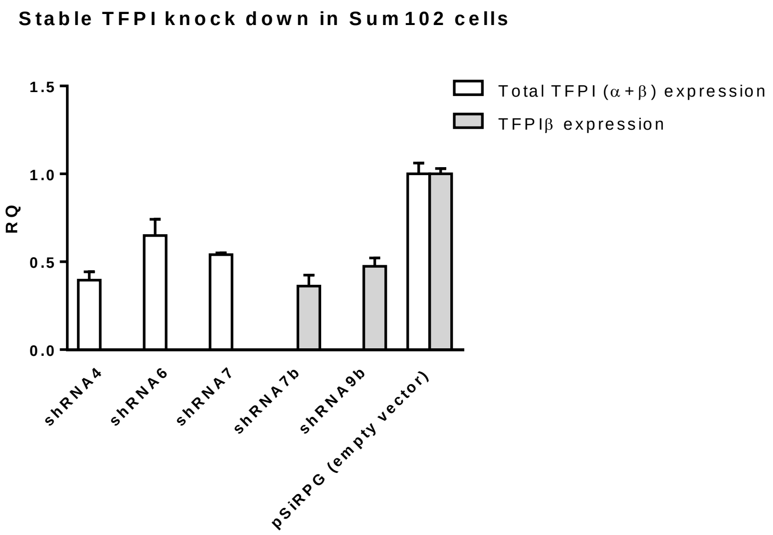
Stable TFPI knock down in Sum102 cells
Total TFPI (α+β) expression
TFPIβ expression
RQ
1.5
1.0
0.5
0.0
shRNA4
shRNA6
shRNA7
shRNA7b
shRNA9b
pSiRPG (empty vector)

B

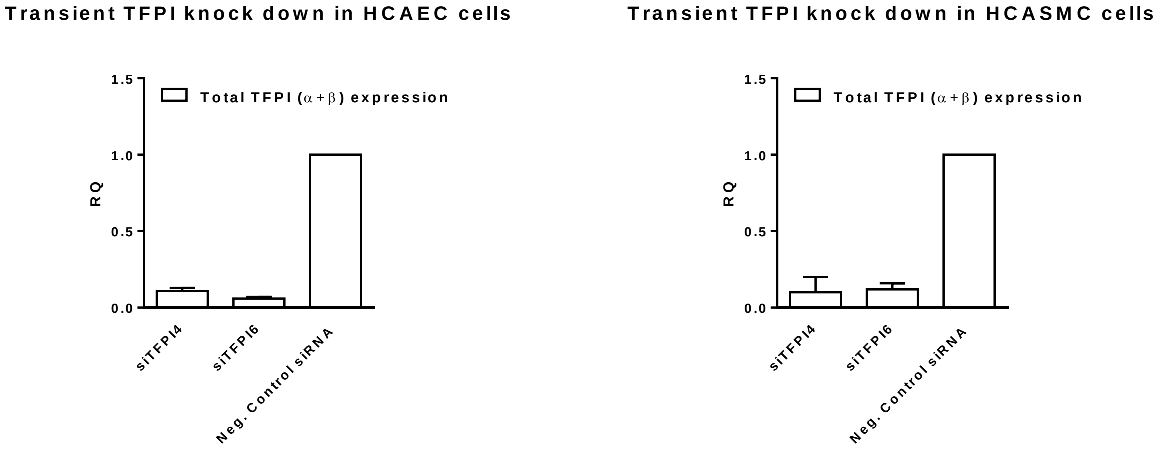
Transient TFPI knock down in HCAEC cells
Total TFPI (α+β) expression
RQ
1.5
1.0
0.5
0.0
siTFPI4
siTFPI6
Neg. Control siRNA
Transient TFPI knock down in HCASMC cells
Total TFPI (α+β) expression
RQ
1.5
1.0
0.5
0.0
siTFPI4
siTFPI6
Neg. Control siRNA

C

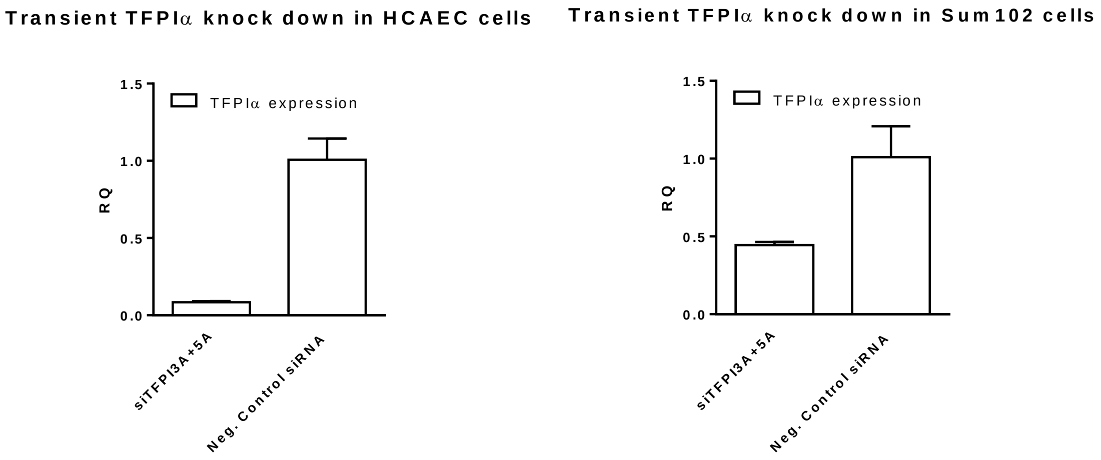
Transient TFPIα knock down in HCAEC cells
TFPIα expression
RQ
1.5
1.0
0.5
0.0
siTFPI3A+5A
Neg. Control siRNA
Transient TFPIα knock down in Sum102 cells
TFPIα expression
RQ
1.5
1.0
0.5
0.0
siTFPI3A+5A
Neg. Control siRNA
