# Supplementary material for: Syndecan-3 and TFPI Colocalize on the Surface of Endothelial-, Smooth Muscle-, and Cancer Cells
Source: PLoS One. 2015 Jan 24;10(1):e0117404. doi: 10.1371/journal.pone.0117404 (PMC4305309; doi:10.1371/journal.pone.0117404)
Supplement: S1 Table — (PDF) [file pone.0117404.s006.pdf]

Table S1. Sequences of siRNA directed against syndecans.

siRNA	Target mRNA	Sequence 5'→ 3'	
SDC1	Syndecan 1	Sense	CACCAUUCUGACUCGGUUUCU
		Antisense	AGAAACCGAGUCAGAAUGGUG
SDC2	Syndecan 2	Sense	CCAUCCAGUGCUGCUUAUC
		Antisense	GAUAAGCAGCACUGGAUGG
SDC3	Syndecan 3	Sense	CCAUACCUGUCCUGAGUUC
		Antisense	GAACUCAGGACAGGUAUGG
SDC4	Syndecan 4	Sense	GUUGUCCAUCCCUUGGUGC
		Antisense	GCACCAAGGGAUGGACAAC
