# Supplementary material for: Syndecan-3 and TFPI Colocalize on the Surface of Endothelial-, Smooth Muscle-, and Cancer Cells
Source: PLoS One. 2015 Jan 24;10(1):e0117404. doi: 10.1371/journal.pone.0117404 (PMC4305309; doi:10.1371/journal.pone.0117404)
Supplement: S2 Table — (PDF) [file pone.0117404.s007.pdf]

Table 2. Probe ID and primer sequences of syndecans (SDC).

Gene Probe	ID*	Forward primer 5’→ 3’	Reverse primer 5’→ 3’
SDC-1	#66	aggatggaggtccttctgc	ccgaggtttcaaaggtgaagt
SDC-2	#22	aaacggacagaagtcctagcag	aaattgcaaagagaaagccaat
SDC-3	#15	tgaactggatgacctctactcg	cgcatggctgtctcaatg
SDC-4	#21	ggcaggaatctgatgactttg	ggccgatcatggagtcttc

* Probes from The Universal Probe Library (Roche Applied Sciences)
